# The importance of conserved amino acids in heme-based globin-coupled diguanylate cyclases

**DOI:** 10.1371/journal.pone.0182782

**Published:** 2017-08-08

**Authors:** Xuehua Wan, Jennifer A. Saito, James S. Newhouse, Shaobin Hou, Maqsudul Alam

**Affiliations:** 1 Department of Microbiology, University of Hawaii, Honolulu, Hawaii, United States of America; 2 Advanced Studies in Genomics, Proteomics and Bioinformatics, University of Hawaii, Honolulu, Hawaii, United States of America; National Renewable Energy Laboratory, UNITED STATES

## Abstract

Globin-coupled diguanylate cyclases contain globin, middle, and diguanylate cyclase domains that sense O_2_ to synthesize c-di-GMP and regulate bacterial motility, biofilm formation, and virulence. However, relatively few studies have extensively examined the roles of individual residues and domains of globin-coupled diguanylate cyclases, which can shed light on their signaling mechanisms and provide drug targets. Here, we report the critical residues of two globin-coupled diguanylate cyclases, *Ec*GReg from *Escherichia coli* and *Bpe*GReg from *Bordetella pertussis*, and show that their diguanylate cyclase activity requires an intact globin domain. In the distal heme pocket of the globin domain, residues Phe42, Tyr43, Ala68 (*Ec*GReg)/Ser68 (*Bpe*GReg), and Met69 are required to maintain full diguanylate cyclase activity. The highly conserved amino acids His223/His225 and Lys224/Lys226 in the middle domain of *Ec*GReg/*Bpe*GReg are essential to diguanylate cyclase activity. We also identified sixteen important residues (Leu300, Arg306, Asp333, Phe337, Lys338, Asn341, Asp342, Asp350, Leu353, Asp368, Arg372, Gly374, Gly375, Asp376, Glu377, and Phe378) in the active site and inhibitory site of the diguanylate cyclase domain of *Ec*GReg. Moreover, *Bpe*GReg_266_ (residues 1–266) and *Bpe*GReg_296_ (residues 1–296), which only contain the globin and middle domains, can inhibit bacterial motility. Our findings suggest that the distal residues of the globin domain affect diguanylate cyclase activity and that *Bpe*GReg may interact with other c-di-GMP-metabolizing proteins to form mixed signaling teams.

## Introduction

Globin-coupled diguanylate cyclases (GCDCs) form a subfamily of globin-coupled sensors (GCS) that are heme-binding sensors linked to variable signaling domains [1–5]. The N-terminal globin domains of GCDCs consist of eight alpha helices and display a myoglobin-like topology [6, 7]. The ferrous ion centered in the heme reversely binds O_2_, and distal residues facilitate O_2_ migration and stabilization in the hydrophobic heme pocket [6, 8–10]. In the C-terminal diguanylate cyclase (DGC) domains of GCDCs, the highly conserved GGD/EEF (Gly-Gly-Asp/Glu-Glu-Phe) motif serves as the active site to synthesize the second messenger bis-(3'-5')-cyclic diguanosine monophosphate (c-di-GMP) [6, 8].

C-di-GMP-dependent signaling pathways regulate diverse cellular functions including motility, biofilm formation, virulence, differentiation, and the cell cycle [11]. Various c-di-GMP receptors have been identified in bacteria and the mammalian innate immune system [12–20]. C-di-GMP is synthesized by DGCs (including GCDCs) containing a GGDEF domain and degraded by phosphodiesterases (PDEs) with either an EAL or HD-GYP domain [11]. Multiple DGCs and PDEs are found in most bacteria and are often associated with sensory or regulatory domains that allow them to modulate their activities in response to internal and environmental stimuli [11]. Studies on how DGCs and PDEs sense environmental signals to regulate c-di-GMP levels will shed light on the mechanisms of bacterial behavior and provide potential drug targets to attenuate the virulence of pathogens.

Various GCDCs have been characterized from *Escherichia coli*, *Bordetella pertussis*, *Azotobacter vinelandii*, *Desulfotalea psychrophila*, *Shewanella putrefaciens*, and *Pectobacterium carotovorum* [6, 8–10, 21–23]. Two GCDCs, *Ec*GReg (also named DosC) from *E*. *coli* and *Bpe*GReg from the whooping cough pathogen *B*. *pertussis*, can sense O_2_ to regulate c-di-GMP synthesis [6, 8]. They differ in respect of O_2_ affinity and cooperative PDEs [6, 8, 24]. *Ec*GReg and *Ec* DosP (EAL type) couple to control c-di-GMP homeostasis, whereas *Bpe*GReg may cooperate with *Bpe* RpfG (HD-GYP type) [8, 24]. To address related questions on how globins regulate DGC activity, here we report the critical residues identified in the three domains of GCDCs and that GCDCs require an intact globin domain for their enzyme activities. We examined Phe42, Tyr43, Ala68 (*Ec*GReg)/Ser68 (*Bpe*GReg), and Met69, which are in the distal heme pocket of the globin domain. We also tested the highly conserved amino acids His223/His225 and Lys224/Lys226 in the middle domain of *Ec*GReg and *Bpe*GReg. In addition, we used *Ec*GReg as a model to examine sixteen conserved residues in the active site and inhibitory site of the DGC domains. We propose that distal globin residues facilitate O_2_ binding to regulate DGC activity and *Bpe*GReg may interact with other DGCs to form mixed signaling teams.

## Materials and methods

### Sequence alignment and visualization of predicted critical residues

Protein sequences were aligned using EBI-MAFFT [25, 26]. The alignment was visualized by using EBI-MView [26, 27]. The homology model of *Bpe*GReg was created previously [6]. Visualization of the locations of the critical residues was carried out using UCSF Chimera [28].

### Plasmid construction

The *ecGReg* (GenBank accession no. NP_416007) and *bpeGReg* (GenBank accession no. NP_882025) genes were cloned into the pTrc99A vector (primers listed in Table A in S1 File). The truncated versions of these genes were also cloned into the pTrc99A vector (primers listed in Tables B and C in S1 File).

The plasmids containing the full-length genes were used as templates for the QuickChange site-directed mutagenesis protocol (Stratagene). Briefly, *PfuTurbo* DNA polymerase (Stratagene) and the primers listed in Tables B and C in S1 File were used for polymerase chain reactions (PCR). Thermal cycling was carried out with 12 cycles as follows: 94°C for 30 seconds, 55°C for 30 seconds, and 68°C for 12 minutes, with a final extension at 68°C for 7 minutes. One microliter of *Dpn*I (10 U/μl, Promega) was then added to digest the methylated parental DNA template at 37°C for 1 hour. The mutated plasmids were transformed into *E*. *coli* TOP10 cells (Invitrogen). The coding regions of the isolated plasmids were verified by Sanger sequencing on ABI 3730xl DNA analyzer.

For protein expression and purification, full-length and truncated *Bpe*GReg proteins were engineered with an N-terminal hexahistidine tag by PCR. Primers are listed in Table D in S1 File. The PCR products were cloned into the pCR4Blunt-TOPO vector (Invitrogen) and then subcloned into the pET-3a expression vector (Novagen).

The laboratory protocol of the molecular cloning has been deposited in protocols.io (DOI: http://dx.doi.org/10.17504/protocols.io.ijxccpn).

### Phenotypic assays

Plasmids were transformed into *Salmonella typhimurium* ATCC 14028 by electroporation and maintained in LB broth or plates with ampicillin (100 μg/ml) at 37°C. For biofilm formation, cells were grown on LB without salt plates containing Congo red (40 μg/ml) for 40 hours at 37°C. Swimming motility was assayed on 0.3% agar plates (1% tryptone, 0.5% NaCl, 1 μM thiamine) at 28°C for 6 hours. Due to leaky expression of the pTrc99A vector, isopropyl β-D-thiogalactopyranoside (IPTG) was not used to induce protein expression during these assays.

### Overexpression, purification and absorption spectra

His-tagged *Bpe*GReg, *Bpe*GReg_155_, *Bpe*GReg_266_, and *Bpe*GReg_296_ were overexpressed in *E*. *coli* Rosetta2(DE3)pLysS cells for 6–8 h at room temperature. Protein expression was induced with 0.05 mM isopropyl-β-D-thiogalactopyranoside. Proteins were purified by Co^2+-^affinity chromatography according to Piatibratov et al. [29]. The detailed protocol has been deposited in protocols.io (DOI: http://dx.doi.org/10.17504/protocols.io.ikhcct6). Spectra were measured using a Cary 1E UV-Visible spectrophotometer (Varian).

## Results and discussion

### Prediction of critical residues of globin-coupled diguanylate cyclases

**Fig 1:**
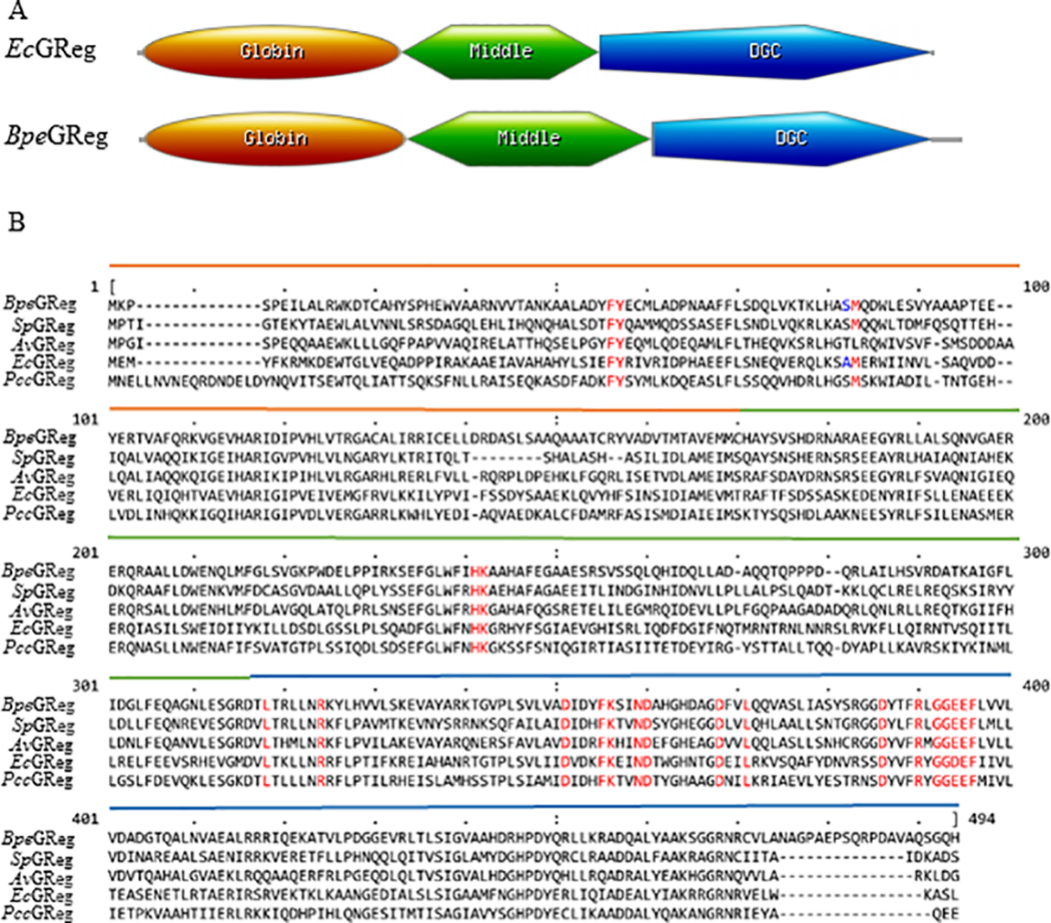
Two globin-coupled diguanylate cyclases consist of three domains. (A) Domain structures of *Ec*GReg and *Bpe*GReg. BLASTP searches against the non-redundant protein sequences database at the National Center for Biotechnology Information showed that *Ec*GReg residues 5–153 represent a globin sensor domain and residues 268–458 represent a DGC domain, while *Bpe*GReg residues 5–155 and residues 297–457 represent a globin sensor domain and a DGC domain, respectively. (B) Amino acid sequence alignment of five globin-coupled diguanylate cyclases: *Ec*GReg, *Bpe*GReg, *Av*GReg (*Azotobacter vinelandii*), *Sp*GReg (*Shewanella putrefaciens*), and *Pcc*GReg (*Pectobacterium carotovorum*). The sequence order is based on the alignment. The residues highlighted in red are highly conserved and discussed in this work. The residues highlighted in blue are not highly conserved and discussed in this work. The line colors indicate the globin (orange), middle (green), and DGC (blue) domains.

**Fig 2:**
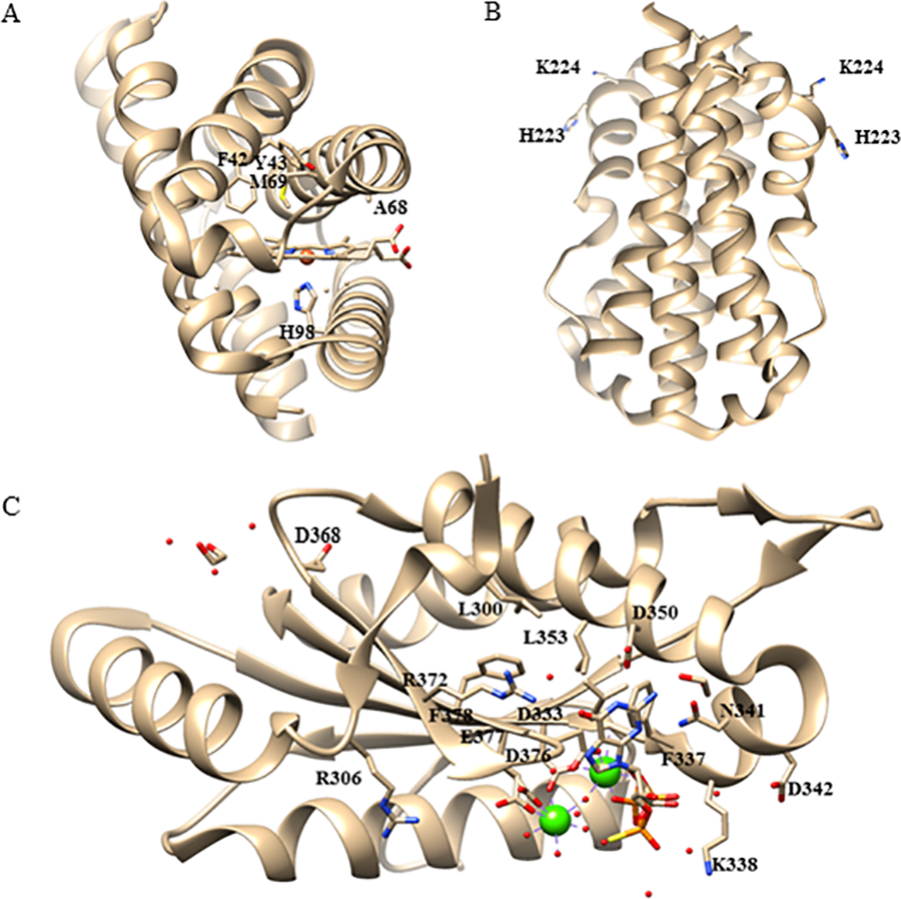
Positions of predicted critical amino acids in the crystal structure of *Ec*GReg/DosC. (A) Crystal structure of the globin domain of *Ec*GReg/DosC (pdb: 4zvb, ferrous form) shows the locations of the critical residues in the heme pocket. H98 is the proximal histidine which binds the heme. The side chains of F42, Y43, and M69 surround the heme center. The side chain of A68 points away from the heme center. (B) Crystal structure of the middle domain of *Ec*GReg/DosC (pdb: 4zvc, form I) shows the locations of critical residues H223 and K224, (C) Crystal structure of the DGC domain of *Ec*GReg/DosC (pdb: 4zvf, GTPαS-bound) shows the locations of mutated residues in this work.

The open reading frames of *ecGReg* and *bpeGReg* encode multidomain proteins containing 460 and 475 amino acids, respectively (Fig 1A). The region linking the N-terminal globin domain and C-terminal DGC domain could not be classified but is highly conserved in many GCDCs, including *Ec*GReg and *Bpe*GReg [3]. We refer to this region as the middle domain (Fig 1A). Fig 1B shows the protein sequence alignment of *Ec*GReg, *Bpe*GReg, and other GCDCs with the same domain architecture. We predicted a series of residues in the globin, middle, and DGC domains of *Ec*GReg and *Bpe*GReg that may affect their enzymatic activities, based on amino acid sequence alignment and the homology model of *Bpe*GReg [6]. The crystal structure of *Ec*GReg [7] was not available at the time this study was performed, so it was only used for subsequently visualizing the positions of the residues we tested rather than aiding our predictions (Fig 2).

In the *Ec*GReg globin domain, the distal residue Tyr43 stabilizes O_2_ to ferrous ion [10]. Our unpublished data suggests a similar role for Tyr43 in *Bpe*GReg. We selected Tyr43 and three other residues in the distal heme pocket of both proteins for site-directed mutagenesis. In the middle domain, *Bpe*GReg His225 was examined previously [6]. Here we selected His223 in *Ec*GReg and nearby conserved residues Lys224 (*Ec*GReg)/Lys226 (*Bpe*GReg) for mutagenesis. To analyze the conserved residues in the DGC domain, we used *Ec*GReg as a model and selected 16 residues in the active and inhibitory sites.

**Fig 3:**
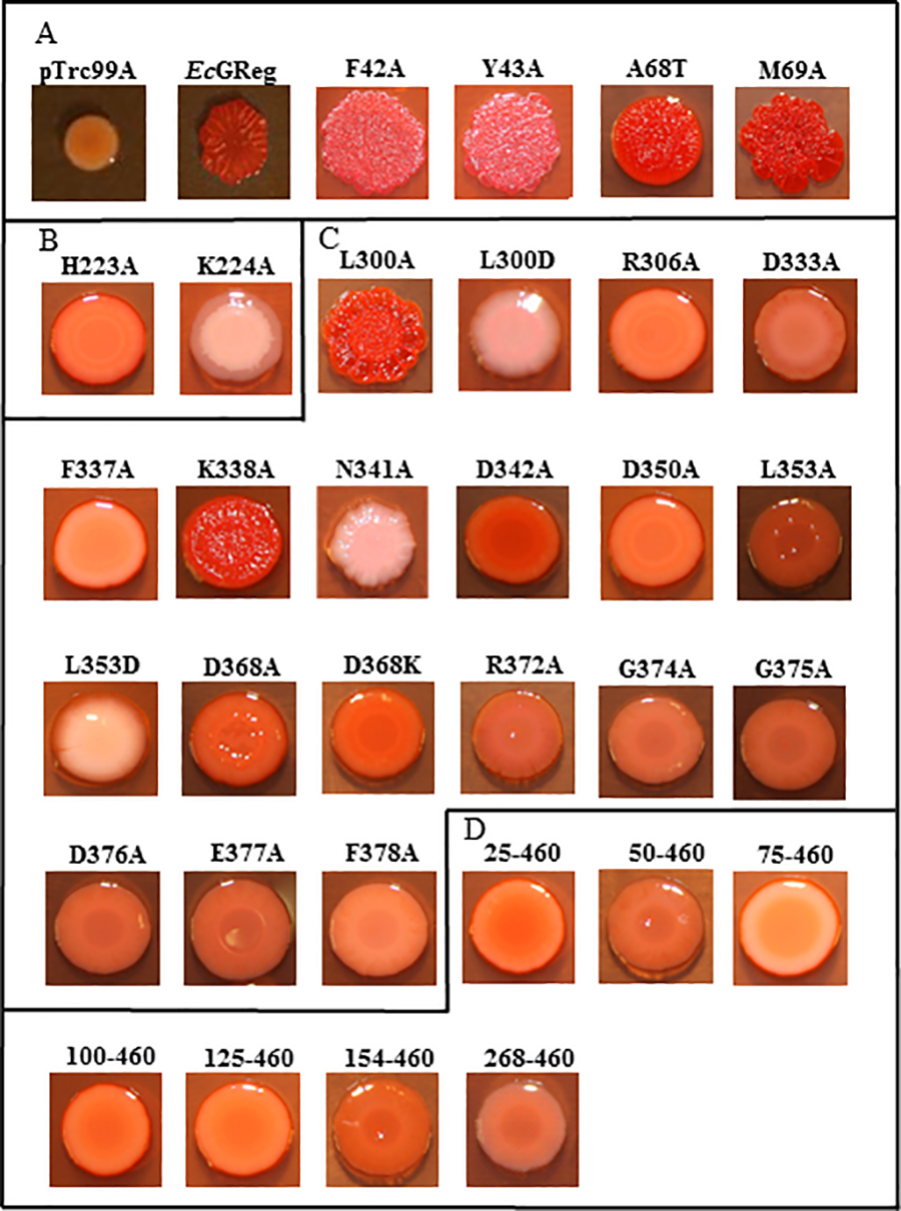
Rdar morphotype development of *Ec*GReg mutants on Congo red agar plates. (A) Rdar morphotypes of *Ec*GReg globin domain mutants. (B) Rdar morphotypes of *Ec*GReg middle domain mutants. (C) Rdar morphotypes of *Ec*GReg DGC domain mutants. (D) Rdar morphotypes of truncated *Ec*GReg proteins.

**Fig 4:**
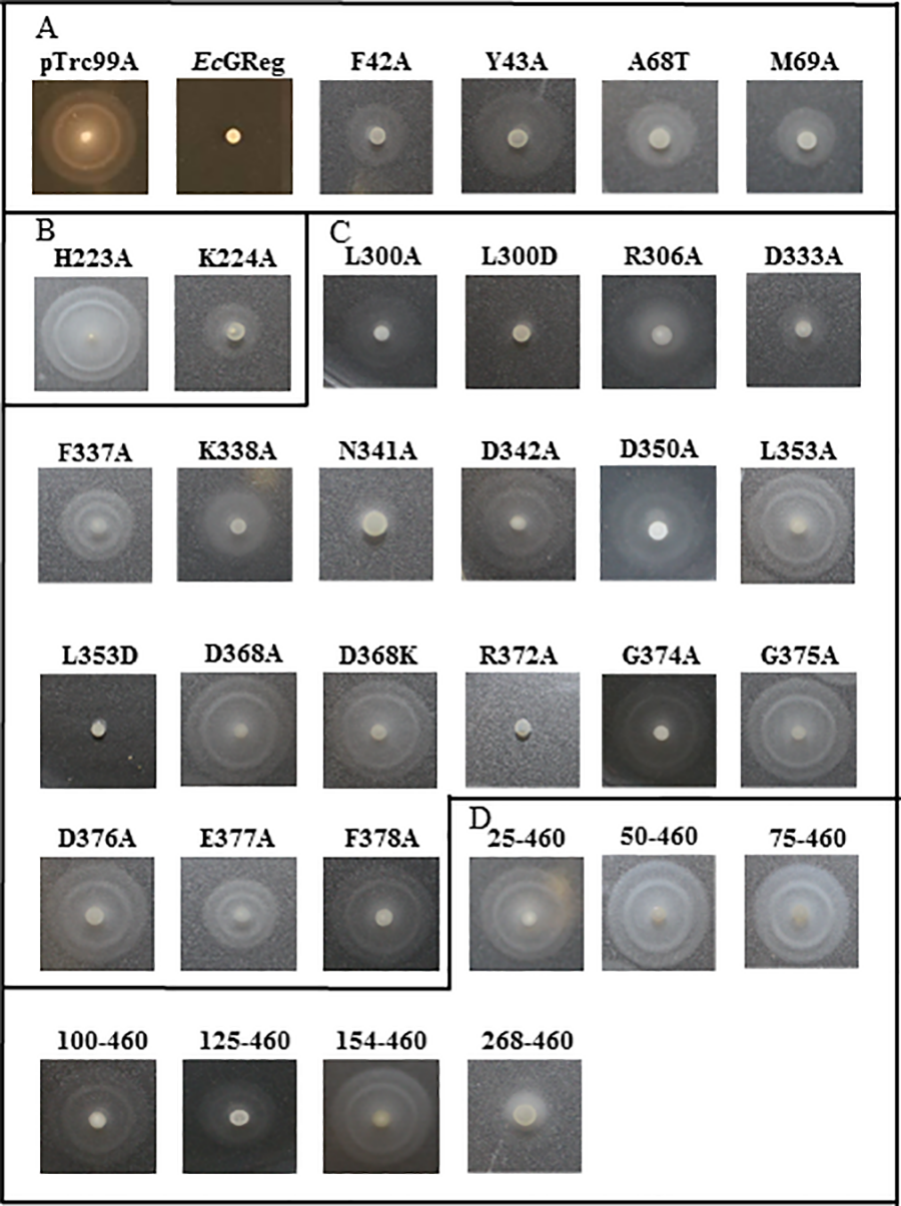
Swimming motility of *Ec*GReg mutants on 0.3% tryptone agar plates. (A) Swimming motility of *Ec*GReg globin domain mutants. (B) Swimming motility of *Ec*GReg middle domain mutants. (C) Swimming motility of *Ec*GReg DGC domain mutants. (D) Swimming motility of truncated *Ec*GReg proteins.

**Fig 5:**
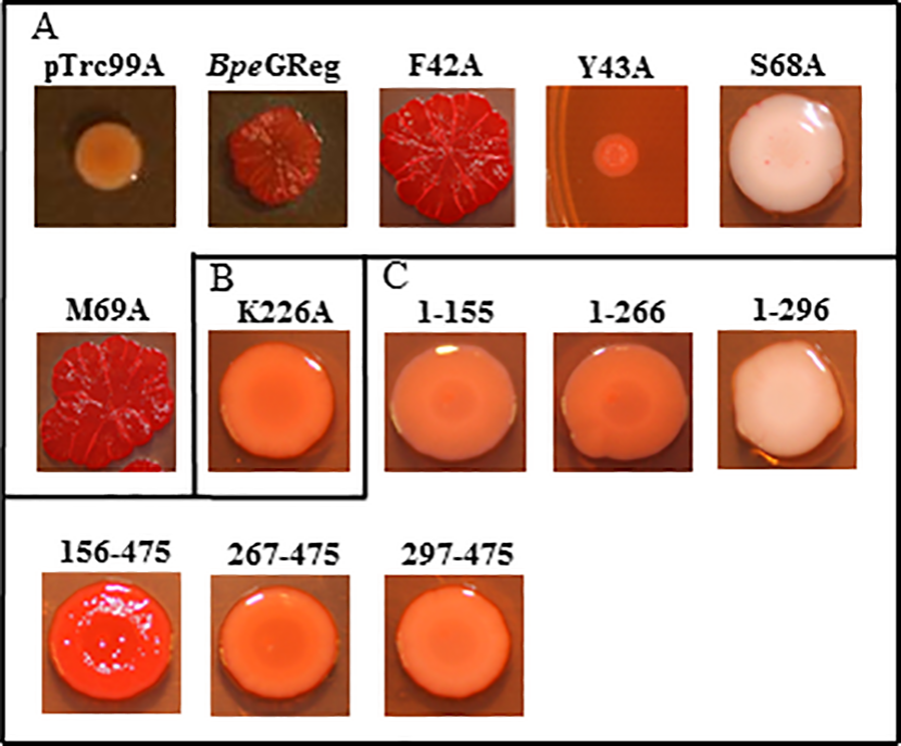
Rdar morphotype development of *Bpe*GReg mutants on Congo red agar plates. (A) Rdar morphotypes of pTrc99A vector control, *Bpe*GReg, and *Bpe*GReg globin domain mutants. (B) Rdar morphotypes of *Bpe*GReg middle domain mutant. (C) Rdar morphotypes of truncated *Bpe*GReg proteins.

**Fig 6:**
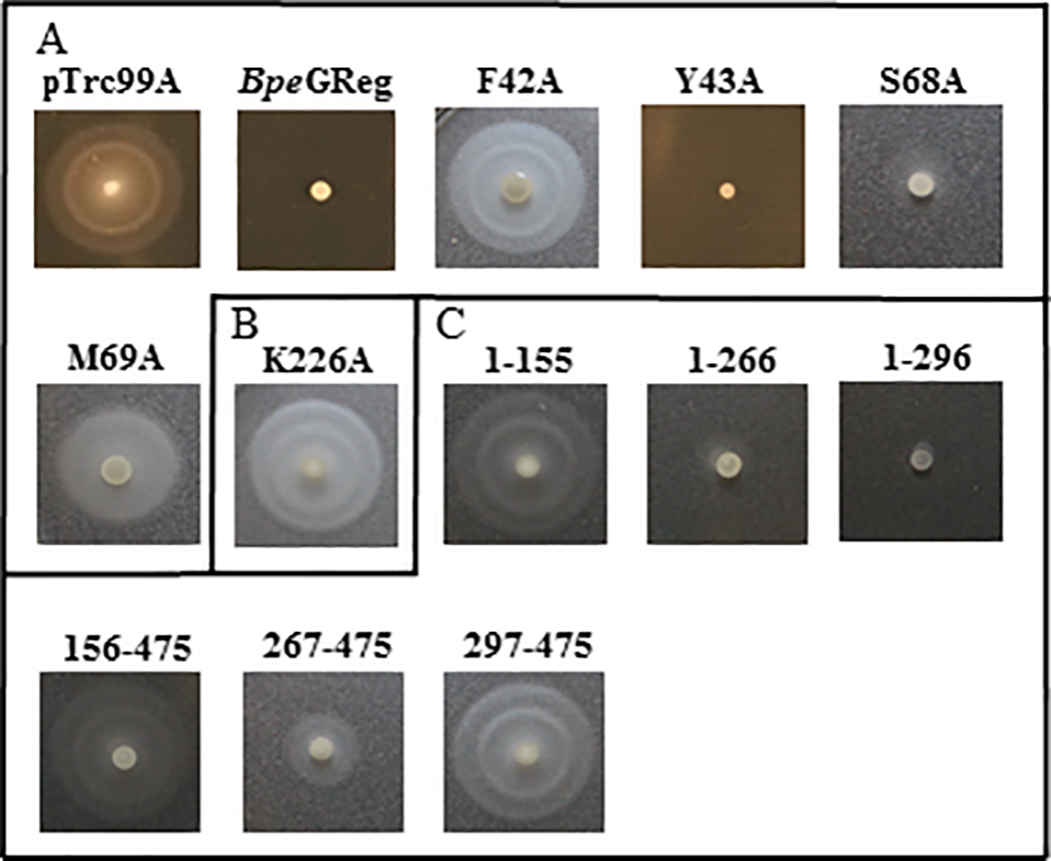
Swimming motility of *Bpe*GReg mutants on 0.3% tryptone agar plates. (A) Swimming motility of pTrc99A vector control, *Bpe*GReg, and *Bpe*GReg globin domain mutants. (B) Swimming motility of *Bpe*GReg middle domain mutant. (C) Swimming motility of truncated *Bpe*GReg proteins.

Mutated proteins were expressed in *Salmonella typhimurium* ATCC 14028 to examine their *in vivo* DGC activities by rdar (red, dry, and rough) formation and motility assays (Figs 3–6, Tables 1 and 2). High levels of intracellular c-di-GMP can inhibit motility, increase the production of exopolysaccharides (EPS) (*e*.*g*., cellulose) and adhesion factors *(e*.*g*., curli fimbriae), and enhance biofilm formation [30]. *S*. *typhimurium* develops an rdar morphotype on Congo red agar plates at 28°C but not at 37°C, indicating the expression of cellulose and curli fimbriae [31, 32]. At 37°C, the temperature regulation of rdar morphotype development can be overcome by overexpression of the DGC AdrA as well as heterologous expression of other DGCs such as *Ec*GReg and *Bpe*GReg [6, 30, 33].

**Table 1:** Summary of phenotypes of *S*. *typhimurium* expressing *Ec*GReg mutants.

Residue	Mutant	RDAR	Motility inhibition	Group
**Globin domain**				
F42	A	+	-	2
Y43	A	+	-	2
A68	T	partial	-	2
M69	A	+	-	2
**Middle domain**				
H223	A	-	-	1
K224	A	-	-	1
**DGC domain**				
L300	A	partial	-	2
	D	-	+	3
R306	A	-	-	1
D333	A	-	-	1
F337	A	-	-	1
K338	A	partial	-	2
N341	A	-	partial	3
D342	A	-	-	1
D350	A	-	-	1
L353	A	-	-	1
	D	-	+	3
D368	A	-	-	1
	K	-	-	1
R372	A	-	+	3
G374	A	-	-	1
G375	A	-	-	1
D376	A	-	-	1
E377	A	-	-	1
F378	A	-	-	1

**Table 2:** Summary of phenotypes of *S*. *typhimurium* expressing *Bpe*GReg mutants.

Residue	Mutant	RDAR	Motility inhibition	Group
**Globin domain**				
F42	A	+	-	2
Y43	A	partial	+	3
S68	A	-	-	1
M69	A	+	-	2
**Middle domain**				
K226	A	-	-	1

### Phe42, Tyr43, Ala68/Ser68 and Met69 in the globin distal heme pockets of *Ec*GReg and *Bpe*GReg are required for DGC activity

In the N-terminal globin domains, we selected residues because they were located near the distal heme pocket, affecting the orientation and migration of ligands. Expression of the *Ec*GReg distal residue mutants, F42A, Y43A, A68T, and M69A, produced the rdar morphotype but failed to inhibit *S*. *typhimurium* motility (Figs 3A and 4A). Similar results were also observed for the *Bpe*GReg mutants F42A and M69A (Figs 5A and 6A). On the other hand, the *Bpe*GReg Y43A mutation severely impaired rdar development but did not affect the ability to inhibit motility (Figs 5A and 6A). Only the *Bpe*GReg S68A mutant could neither confer the rdar morphotype nor inhibit motility (Figs 5A and 6A). These results indicate that residues in the heme pocket of the globin domain are required for *Ec*GReg and *Bpe*GReg to be fully active. Particularly, in *Bpe*GReg, the hydroxyl group of Ser68 is required for full protein function.

### His223/His225 and Lys224/Lys226 in the middle domains of *Ec*GReg and *Bpe*GReg are required for DGC activity

We previously demonstrated that *Bpe*GReg could be inactivated by an H225A mutation in the middle domain, as no detectable c-di-GMP was produced *in vitro* and no c-di-GMP-dependent physiological effects were observed when expressed in *S*. *typhimurium* [6]. When the corresponding residue in *Ec*GReg was mutated (H223A), this protein was also unable to induce the rdar morphotype or inhibit motility in *S*. *typhimurium* (Figs 3B and 4B). Mutation of another highly conserved residue, K224A (*Ec*GReg)/K226A (*Bpe*GReg), led to inactive phenotypes as well (Figs 3B–6B). Taken together, these results suggest an essential role of the middle domain for DGC activation. *Ec*GReg His223 and Lys224 locate in a π-helix (residues 221–225) that constitutes the middle region of the α-helix B (residues 216–228) of the middle domain [7]. Although the α-helices-associated π-helices are overlooked and rarely annotated, they are present in ~15% of all proteins and tend to be associated with protein function, *e*.*g*. peristaltic-like shifts to extend the binding cavity of a substrate [34]. In the homodimer structure of the *Ec*GReg middle domain (Fig 2B) [7], the side chains of His223 and Lys224 point towards the surrounding environment, contributing to the positive charges on the protein surface. Charged side chains on protein surfaces may play roles in allosteric regulation, protein-protein interaction, or folding/stability of protein. Further investigation will help us to understand the specific roles of the middle domain and the conserved π-helix of GCDCs.

### Critical residues in the A-site and I-site of the DGC domain are required for enzyme activity

DGCs function as homodimers, with the two monomers forming the active site (A-site) at the dimer interface [35, 36]. The signature GG(D/E)EF motif constitutes part of the A-site, and several studies have suggested that absolute conservation of all five residues in this motif is required for catalysis [37, 38]. However, the first residue of the GG(D/E)EF motif appears to be flexible to substitutions in some DGCs. A DGC from *Pectobacterium atrosepticum* was observed to be active with Gly, Ser, or Ala in the first position, while a DGC from *Vibrio cholerae* was active with Gly, Ala, Met, or His [39, 40]. Using *Ec*GReg as a model, we wanted to determine which residues of the GGDEF motif were essential for its catalytic function. We therefore mutated each residue of the GGDEF motif to Ala (G374A, G375A, D376A, E377A, and F378A). All of these mutants failed in conferring the rdar morphotype or inhibiting motility in *S*. *typhimurium* (Figs 3C and 4C), indicating that the canonical GGDEF motif is required for the DGC activity of *Ec*GReg. We further examined the roles of other residues within the A-site. Six mutations (R306A, D333A, F337A, D342A, D350A, and L353A) resulted in a lack of *in vivo* function (Figs 3C and 4C). Six other mutants retained partial function: cells harboring the L300A and K338A mutants formed a partial rdar morphotype (Fig 3C), while the L300D, N341A, L353D, and R372A mutants were able to inhibit or partially inhibit motility (Fig 4C). These results highlight the requirement of additional A-site residues for DGC activity, besides those of the GGDEF motif. Many of the residues (Asp333, Phe337, Lys338, Asn341, and Asp350) directly interact with either the substrate or metal ions in the A-site [7]. The other residues may help maintain the functional structure of the DGC domain.

An inhibitory site (I-site), consisting of an R*XX*D motif, is found in DGC domains, including those of *Ec*GReg and *Bpe*GReg [41]. The I-site is located five residues upstream of the GG(D/E)EF motif (Fig 1B). Feedback inhibition occurs when a c-di-GMP dimer binds to this allosteric site, thus decreasing DGC activity [35, 41]. We tested the effect of mutating the conserved Asp in the R_365_*XX*D_368_ motif of *Ec*GReg. The D368A and D368K variants both lacked the ability to produce the rdar morphotype or inhibit motility in *S*. *typhimurium* (Figs 3C and 4C). This is consistent with the findings of Kitanishi *et al*. [10], who observed that *Ec*GReg D368A did not have biofilm formation activity when expressed in *E*. *coli*. Furthermore, Burns *et al*. [42] found that mutation of the first Arg in the *Bpe*GReg I-site (R364A) affected oligomerization and decreased catalytic activity, leading to the conclusion that the I-site plays a role in controlling the conformation/dynamics of DGC domains, in addition to product inhibition.

The mutations characterized in this study can be classified into three groups: 1) mutants that could no longer confer the rdar morphotype or inhibit motility; 2) mutants that could confer the rdar morphotype but not inhibit motility; and 3) mutants that could inhibit motility but not confer the rdar morphotype (Tables 1 and 2). We conclude that the group 1 mutants, which are mainly located within the middle and DGC domains, include the most crucial residues required for DGC activity. The group 2 and 3 mutants are found in the globin and DGC domains. Particularly, the globin distal residue mutants Y43A are classified into different groups (*Ec*GReg in group 2 versus *Bpe*GReg in group 3). The differential effects of the group 2 and 3 mutants on rdar formation and motility could be due to signaling specificity. Rdar formation and motility inhibition involve a number of processes that are regulated at multiple levels, and particular DGCs and/or PDEs are often attributed to regulating specific processes [11]. The molecular mechanisms by which *Ec*GReg and *Bpe*GReg exert their specificities are not yet clearly understood. We presume that the group 2 and 3 mutations led to decreased c-di-GMP production, resulting in threshold levels sufficient for activating one phenotype over the other. This could involve c-di-GMP receptors with different binding affinities [43]. However, we cannot exclude the possibility that proximity to the appropriate targets was disrupted, as some of the mutations may have affected localization of the GCDC proteins in the cell or their association into protein complexes. It was shown that *Ec*GReg, along with *Ec* DosP, associates with PNPase in a ribonucleoprotein complex to regulate RNA turnover [15], but it is unknown how this may be connected to biofilm formation or motility.

The biofilm formation (rdar) activities of a few *Ec*GReg mutant variants have been previously evaluated in *E*. *coli* [10, 44], and the results were, for the most part, in agreement with what we observed for similar mutant proteins expressed in *S*. *typhimurium*. However, there was one discrepancy. While the H223A mutant displayed a wild-type phenotype in *E*. *coli* [10], our result indicated that the activity of the mutant was impaired. Differences in the c-di-GMP signaling networks of *E*. *coli* and *S*. *typhimurium* may account for the different phenotypic outcomes [45]. Contributions of species-specific DGCs/PDEs, interactions with distinct sets of proteins, or differences in the c-di-GMP thresholds needed to elicit a phenotypic response are among the possible factors that could lead to the variable effects seen in these organisms.

### *S*. *typhimurium* expressing individual domains of *Bpe*GReg and *Ec*GReg cannot form the full rdar morphotype, while *Bpe*GReg_266_ and *Bpe*GReg_296_ can inhibit motility

**Fig 7:**
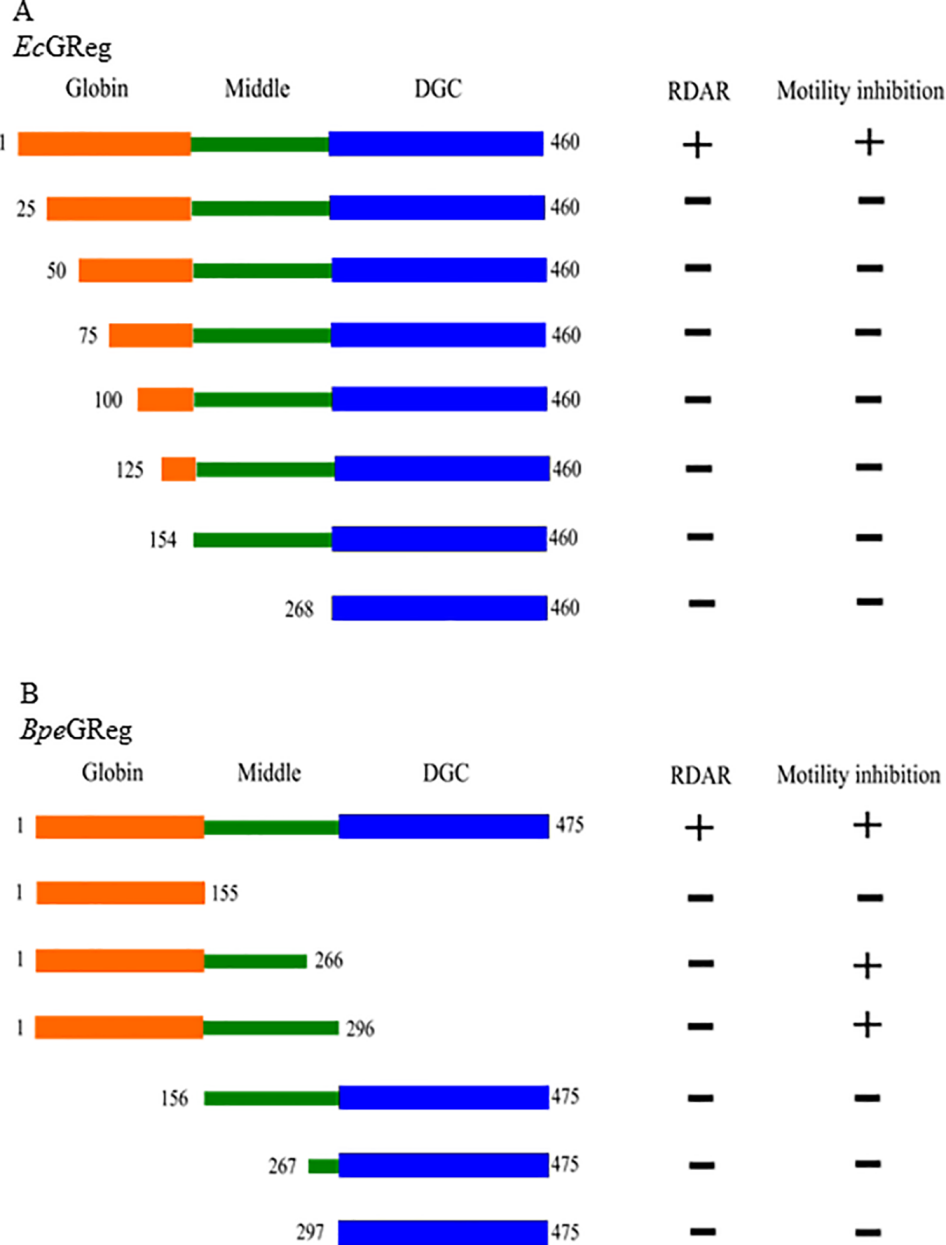
Schematic diagram of truncated *Ec*GReg (A) and *Bpe*GReg (B).

Individual DGC domains have been shown to possess low-level enzymatic activity *in vitro*, which is several orders of magnitude lower than that of the full-length proteins [46]. A construct containing the middle and DGC domains of *Ec*GReg (residues 173–460) exhibited four-fold less activity than the full-length protein, whereas the DGC domain alone (residues 297–460) did not have any detectable activity due to it being monomeric in solution [7]. In order to assess whether truncated forms of *Ec*GReg and *Bpe*GReg have DGC activity *in vivo*, we tested the functions of various constructs in *S*. *typhimurium* (Fig 7). We used *Ec*GReg as a model to examine whether it could maintain its function when the globin domain was gradually truncated. All of the *Ec*GReg truncated proteins could not confer a full rdar morphotype (Fig 3D), and were unable to inhibit motility as well (Fig 4D). We also tested the individual or combined domains of *Bpe*GReg. Intriguingly, two of the *Bpe*GReg constructs, *Bpe*GReg_266_ and *Bpe*GReg_296_ (residues 1–266 and 1–296), which only contain the globin and middle domains, were able to inhibit motility (Fig 6C). Compared to wild-type *Bpe*GReg, the absorption spectra of *Bpe*GReg_155_, *Bpe*GReg_266_ and *Bpe*GReg_296_ showed that they were able to bind heme (Fig A in S1 File), indicating that the heme-based globin was present. The presence of the globin domain alone (*Bpe*GReg_155_), however, was not sufficient for eliciting a physiological response. Our data suggest that both the heme-based globin and middle domain are required for signaling and they may interact with other DGCs to form signaling clusters.

Our analysis of the truncated proteins suggests that the complete structures of *Ec*GReg and *Bpe*GReg are required for full DGC activity. Previous studies have demonstrated that the cyclase activity of GCDCs is regulated by O_2_ binding to the globin sensor domain [6, 8, 21–23], so it is not surprising that the presence of this domain would be necessary for optimal function. However, it is unexpected to find that expression of two *Bpe*GReg constructs lacking the DGC domain resulted in motility inhibition. As these proteins did not have the ability to synthesize c-di-GMP, we postulate that the globin and middle domains of *Bpe*GReg may be able to interact with other DGCs, PDEs, or effector proteins to coordinate c-di-GMP signaling. Thus, the function of the truncated *Bpe*GReg proteins may be partially rescued by its partners in mixed signaling teams. There is growing evidence for the involvement of protein-protein interactions in regulating c-di-GMP networks. For example, *Ec*GReg forms a complex with the PDE DosP [8]; the stand-alone NO sensor H-NOX binds to HaCE, a dual-functioning DGC and PDE in *Shewanella woodyi* [47, 48]; and the DGC GcbC associates with its target receptor LapD in *Pseudomonas fluorescens* [49, 50].

## Conclusion

Characterization of *Ec*GReg and *Bpe*GReg will provide general insights into the structure and function of GCSs. Here we provided evidence that residues Phe42, Tyr43, Ala68/Ser68 and Met69 in the distal heme pockets of *Ec*GReg and *Bpe*GReg could affect C-terminal DGC activity. His223/His225 and Lys224/Lys226 in the middle domains of *Ec*GReg and *Bpe*GReg were also required, and a number of critical residues in the A-site and I-site of the *Ec*GReg DGC domain were identified. In addition, the full globin fold is required for GCDC activity. We further hypothesize that *Bpe*GReg, via its globin and middle domains, may be able to form clusters with other c-di-GMP-metabolizing proteins. This may shed light on the functions of other globins, especially those single-domain proteins with unknown functions.

## Supporting information

S1 File **Table A. Primers used for cloning *Ec*GReg and *Bpe*GReg in pTrc99A. Table B. Primers used for the construction of *Ec*GReg mutants and truncated *Ec*GReg. Table C. Primers used for the construction of *Bpe*GReg mutants and truncated *Bpe*GReg. Table D. Primers used for expression of truncated *Bpe*GReg in pET-3a. Figure A. Absorption spectra of truncated *Bpe*GReg proteins.** a) Wild-type *Bpe*GReg (solid red line) showed heme-bound absorption spectra. b) *Bpe*GReg_155_, *Bpe*GReg_266_, and *Bpe*GReg_296_ showed heme-bound absorption spectra, similar to that of wild-type *Bpe*GReg.
(PDF)
