# Supplementary material for: The importance of conserved amino acids in heme-based globin-coupled diguanylate cyclases
Source: PLoS One. 2017 Aug 8;12(8):e0182782. doi: 10.1371/journal.pone.0182782 (PMC5549716; doi:10.1371/journal.pone.0182782)

Supporting Information – S1 File

Table A. Primers used for cloning *Ec*GReg and *Bpe*GReg in pTrc99A.

Primer name	Primer sequence (5'-3')
*Ec*GReg SacI top	AAGAGCTCATGGAGATGTATTTTAAAAG
*Ec*GReg BamHI bot	AAGGATCCCTAAAGACTGGCTTTCCAG
*Bpe*GReg SacI top	AAGAGCTCTTGAAGCCTTCGCCTGAAATC
*Bpe*GReg BamHI bot	AAGGATCCCTAGTGCTGGCCGGACTG

Table B. Primers used for the construction of *Ec*GReg mutants and truncated *Ec*GReg.

Primer Name	Primer Sequence 5'-3'
**Primers for globin domain mutants**	
*Ec*GReg F42A mutF	CATTATCTGAGTATCGAGGCCTATCGAATTGTCCGC
*Ec*GReg F42A mutR	GCGGACAATTCGATAGGCCTCGATACTCAGATAATG
*Ec*GReg Y43A mutF	CTGAGTATCGAGTTTGCCCGAATTGTCCGCATCG
*Ec*GReg Y43A mutR	CGATGCGGACAATTCGGGCAAACTCGATACTCAG
*Ec*GReg A68T mutF	GCGGCAGTTGAAGAGTACCATGGAACGCTGGATTATTAAC
*Ec*GReg A68T mutR	GTTAATAATCCAGCGTTCCATGGTACTCTTCAACTGCCGC
*Ec*GReg M69A mutF	CAGTTGAAGAGTGCGGCCGAACGCTGGATTATTAAC
*Ec*GReg M69A mutR	GTTAATAATCCAGCGTTCGGCCGCACTCTTCAACTG
**Primers for DGC domain mutants**	
*Ec*GReg G374A mutF	GATTATGTTTTCCGCTACGCCGGCGATGAATTTATCATTG
*Ec*GReg G374A mutR	CAATGATAAATTCATCGCCGGCGTAGCGGAAAACATAATC
*Ec*GReg G375A mutF	GTTTTCCGCTACGGGGCCGATGAATTTATCATTG
*Ec*GReg G375A mutR	CAATGATAAATTCATCGGCCCCGTAGCGGAAAAC
*Ec*GReg D376A mutF	GTTTTCCGCTACGGGGGCGCCGAATTTATCATTGTTTTG
*Ec*GReg D376A mutR	CAAAACAATGATAAATTCGGCGCCCCCGTAGCGGAAAAC
*Ec*GReg E377A mutF	CCGCTACGGGGGCGATGCCTTTATCATTGTTTTGAC
*Ec*GReg E377A mutR	GTCAAAACAATGATAAAGGCATCGCCCCCGTAGCGG
*Ec*GReg F378A mutF	CTACGGGGGCGATGAAGCCATCATTGTTTTGACTG
*Ec*GReg F378A mutR	CAGTCAAAACAATGATGGCTTCATCGCCCCCGTAG
*Ec*GReg L300A mutF	GTCGGTATGGATGTAGCCACGAAATTACTTAACC
*Ec*GReg L300A mutR	GGTTAAGTAATTTCGTGGCTACATCCATACCGAC
*Ec*GReg L300D mutF	GTCGGTATGGATGTAGACACGAAATTACTTAACC
*Ec*GReg L300D mutR	GGTTAAGTAATTTCGTGTCTACATCCATACCGAC
*Ec*GReg R306A mutF	GACGAAATTACTTAACGCCCGTTTCCTACCGACTATC
*Ec*GReg R306A mutR	GATAGTCGGTAGGAAACGGGCGTTAAGTAATTTCGTC
*Ec*GReg D333A mutF	GTCAGTGCTGATTATTGCCGTTGATAAATTCAAAG
*Ec*GReg D333A mutR	CTTTGAATTTATCAACGGCAATAATCAGCACTGAC
*Ec*GReg F337A mutF	GATTATTGACGTTGATAAAGCCAAAGAGATCAACGATAC
*Ec*GReg F337A mutR	GTATCGTTGATCTCTTTGGCTTTATCAACGTCAATAATC
*Ec*GReg K338A mutF	GACGTTGATAAATTCGCCGAGATCAACGATACG
*Ec*GReg K338A mutR	CGTATCGTTGATCTCGGCGAATTTATCAACGTC
*Ec*GReg N341A mutF	GATAAATTCAAAGAGATCGCCGATACGTGGGGCCATAAC
*Ec*GReg N341A mutR	GTTATGGCCCCACGTATCGGCGATCTCTTTGAATTTATC
*Ec*GReg D342A mutF	GATAAATTCAAAGAGATCAACGCCACGTGGGGCCATAAC

*Ec*GReg D342A mutR	GTTATGGCCCCACGTGGCGTTGATCTCTTTGAATTTATC
*Ec*GReg D350A mutF	GGCCATAACACTGGTGCCGAAATTCTGCGTAAAG
*Ec*GReg D350A mutR	CTTTACGCAGAATTTCGGCACCAGTGTTATGGCC
*Ec*GReg L353A mutF	CACTGGTGATGAAATTGCCCGTAAAGTCTCTCAGG
*Ec*GReg L353A mutR	CCTGAGAGACTTTACGGGCAATTTCATCACCAGTG
*Ec*GReg L353D mutF	CACTGGTGATGAAATTGACCGTAAAGTCTCTCAGG
*Ec*GReg L353D mutR	CCTGAGAGACTTTACGGTCAATTTCATCACCAGTG
*Ec*GReg D368A muF	CAACGTCCGCAGTAGTGCCTATGTTTTCCGCTAC
*Ec*GReg D368A mutR	GTAGCGGAAAACATAGGCACTACTGCGGACGTTG
*Ec*GReg D368K mutF	CAACGTCCGCAGTAGTAAATATGTTTTCCGCTAC
*Ec*GReg D368K mutR	GTAGCGGAAAACATATTTACTACTGCGGACGTTG
*Ec*GReg R372A mutF	GTAGTGATTATGTTTTCGCCTACGGGGGCGATG
*Ec*GReg R372A mutR	CATCGCCCCCGTAGGCGAAAACATAATCACTAC
**Primers for middle domain mutants**	
*Ec*GReg H223A mutF	GGCCTGTGGTTTAACGCCAAAGGTCGACATTATTTTAG
*Ec*GReg H223A mutR	CTAAAATAATGTCGACCTTTGGCGTTAAACCACAGGCC
*Ec*GReg K224A mutF	CCTGTGGTTTAACCATGCCGGTCGACATTATTTTAG
*Ec*GReg K224A mutR	CTAAAATAATGTCGACCGGCATGGTTAAACCACAGG
**Primers for truncated *Ec*GReg**	
*Ec*GReg 25-end SacI top	CGGAGCTCGCTAAAGCCGCGGAAATTG
*Ec*GReg 50-end SacI top	CGGAGCTCCCGCATGCCGAAGAATTC
*Ec*GReg 75-end SacI top	CGGAGCTCAACGTGCTTTCTGCCCAG
*Ec*GReg 100-end SacI top	CGGAGCTCCGCATAGGAATTCCGGTAG
*Ec*GReg 125-end SacI top	CGGAGCTCTCTTCGGATTATTCCGCC
*Ec*GReg 154-end SacI top	AAGAGCTCATGGCGTTTACCTTTAGTGAC
*Ec*GReg 268-end SacI top	AAGAGCTCATGTTTTTATTACAGATAAG

Table C. Primers used for the construction of *Bpe*GReg mutants and truncated *Bpe*GReg.

Primer name	Primer Sequence 5'-3'
**Primers for *Bpe*GReg globin domain mutants**	
*Bpe*GReg F42A mutF	GGCGCTGGCCGATTATGCCTACGAGTGCATGCTGG
*Bpe*GReg F42A mutR	CCAGCATGCACTCGTAGGCATAATCGGCCAGCGCC
*Bpe*GReg Y43A mutF	GCTGGCCGATTATTTCGCCGAGTGCATGCTGGCCG
*Bpe*GReg Y43A mutR	CGGCCAGCATGCACTCGGCGAAATAATCGGCCAGC
*Bpe*GReg S68A mutF	GACCAAGCTGCATGCCGCCATGCAGGATTGGCTGG
*Bpe*GReg S68A mutR	CCAGCCAATCCTGCATGGCGGCATGCAGCTTGGTC
*Bpe*GReg M69A mutF	CAAGCTGCATGCCTCCGCCCAGGATTGGCTGGAATC
*Bpe*GReg M69A mutR	GATTCCAGCCAATCCTGGGCGGAGGCATGCAGCTTG
**Primers for *Bpe*GReg middle domain mutants**	
*Bpe*GReg K226A mutF	CCTGTGGTTCATCCACGCCGCGGCGCACGCCTTCG
*Bpe*GReg K226A mutR	CGAAGGCGTGCGCCGCGGCGTGGATGAACCACAGG
**Primers for truncated *Bpe*GReg**	
*Bpe*GReg 156-475 Sacl top	AAGAGCTCGCCTACTCGGTGTCGCAC
*Bpe*GReg 267-475 Sacl top	AAGAGCTCATCCTGCACAGCGTGCGC
*Bpe*GReg 297-475 Sacl top	AAGAGCTCGATACGCTGACCCGGCTG
*Bpe*GReg 1-155 tga BamHI bot	AAGGATCCTCAATGGCACATCATCTCGAC
*Bpe*GReg 1-266 tga BamHI bot	AAGGATCCTCAGGCCAGGCGCTGGTCGGG
*Bpe*GReg 1-296 tga BamHI bot	AAGGATCCTCAGCGCCCCGACTCCAGG

Table D. Primers used for expression of truncated *Bpe*GReg in pET-3a.

Primer name	Primer sequence (5'-3')
*Bpe*GReg Xa top	ATCGAGGGAAGGTTGAAGCCTTCGCCTGAAATC
Histidine Xa top	CACCACCACCACCACCACATCGAGGGAAGG
NdeI histidine top	AACATATGCACCACCACCACCACCAC
*Bpe*GReg BamHI bot	AAGGATCCCTAGTGCTGGCCGGACTG
*Bpe*GReg 1-155 tga BamHI bot	AAGGATCCTCAATGGCACATCATCTCGAC
*Bpe*GReg 1-266 tga BamHI bot	AAGGATCCTCAGGCCAGGCGCTGGTCGGG
*Bpe*GReg 1-296 tga BamHI bot	AAGGATCCTCAGCGCCCCGACTCCAGG

Figure A. Absorption spectra of truncated *Bpe*GReg proteins. a) Wild-type *Bpe*GReg (solid red line) showed heme-bound absorption spectra. b) *Bpe*GReg$_{155}$, *Bpe*GReg$_{266}$, and *Bpe*GReg$_{296}$ showed heme-bound absorption spectra, similar to that of wild-type *Bpe*GReg.

a. b.

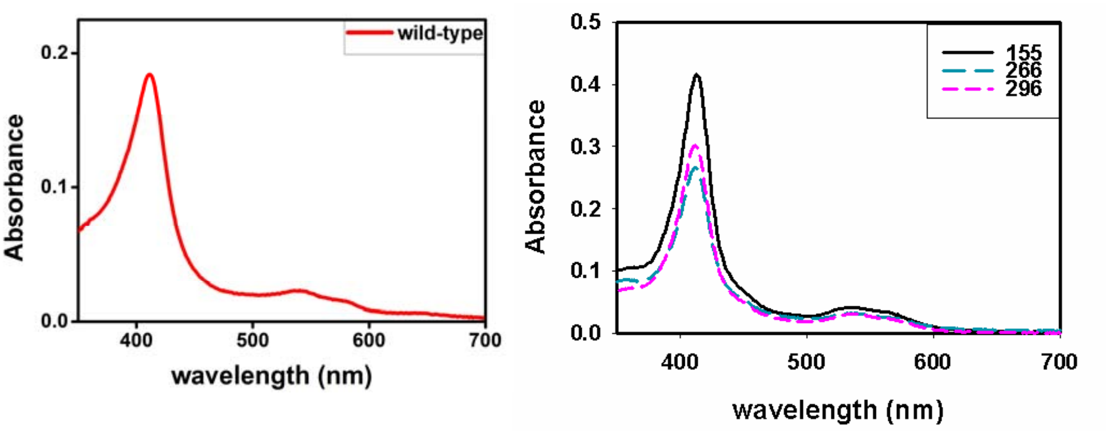

Supplement: S1 File — Table A. Primers used for cloning EcGReg and BpeGReg in pTrc99A. Table B. Primers used for the construction of EcGReg mutants and truncated EcGReg. Table C. Primers used for the construction of BpeGReg mutants and truncated BpeGReg. Table D. Primers used for expression of truncated BpeGReg in pET-3a. Figure A. Absorption spectra of truncated BpeGReg proteins. a) Wild-type BpeGReg (solid red line) showed heme-bound absorption spectra. b) BpeGReg155, BpeGReg266, and BpeGReg296 showed heme-bound absorption spectra, similar to that of wild-type BpeGReg. (PDF) [file pone.0182782.s001.pdf]
